# Effects of stream permanence on stonefly (Insecta, Plecoptera) community structure at Mammoth Cave National Park, Kentucky, USA

**DOI:** 10.3897/BDJ.9.e62242

**Published:** 2021-03-10

**Authors:** Taylor C McRoberts, Scott Grubbs

**Affiliations:** 1 Western Kentucky University, Bowling Green, United States of America Western Kentucky University Bowling Green United States of America

**Keywords:** Plecoptera, stoneflies, Mammoth Cave National Park, stream permanence, perennial, intermittent

## Abstract

Stoneflies (Plecoptera) are often associated with inhabiting cold perennial streams, but many species also inhabit intermittent streams that experience reduced or lack of flow during summer and autumn. In this study, the influence of stream permanence on stonefly assemblage composition and spatial distribution at Mammoth Cave National Park, Kentucky, USA, was addressed, based on a 14 month sampling regime from the fullest range of stream sizes and habitable flow regions available. Adult stoneflies were collected monthly from 43 sites at the Park plus an additional two sites at the near-adjacent Western Kentucky University Green River Preserve. Collections were done from December 2018–November 2019 using a standard timed protocol with beating sheets for adults and once in December 2019–January 2020 for larvae. Stream sites were assigned one of five category types: perennial spring runs, perennial spring seeps, upland perennial streams, perennial riverine and summer dry runs. In total, 34 species were collected. The most prominent difference in stonefly community structure was between spring runs, spring seeps and summer dry streams vs. upland perennial streams. Approximately 88% of species collected had univoltine-fast life cycles and 79% likely had an extended period of egg or larval diapause. Due to the predominance of small upland perennial and summer dry streams, species commonly typically found in larger lotic systems are fundamentally filtered out of the region due to the lack of available habitats. Species able to survive in intermittent habitats do so by life history adaptations including to survive desiccation as larvae or eggs.

## Introduction

Pristine habitats are being degraded around the world at unprecedented rates (Wilson 1989, Sisk et al. 1994). As global human population increases, many habitats are being lost to anthropogenic alterations, including agriculture, urbanisation and forestry practices (Wilson 1989). Protected natural areas, such as National Parks and biological preserves, are widespread across North America and have the potential to offer important refugia to native flora and fauna (Bruner et al. 2001). Knowing the native flora and fauna is important to managers of these protected areas and may help in conservation initiatives (Lomolino 2001). These areas also offer important access for sampling studies and research initiatives (Mitchell and Buggey 2001).

Freshwater systems in protected areas are highly sensitive to many anthropogenic impacts (Ormerod et al. 2010). Climate change brings immense risks for freshwater systems. Increased evaporation rates may lower water levels, reducing suitable habitat for fish and macroinvertebrates (Chu et al. 2005). Increases in water temperature are expected and could alter stratification times of lentic systems globally, leading to decreased suitable habitats for coldwater species and reduced dissolved oxygen concentration (Eaton and Scheller 1996).

Stoneflies (Order Plecoptera) are aquatic insects that are widely distributed across the globe (except Antarctica) and are well known for their ecological roles in lotic ecosystems (Fochetti and Figueroa 2008). Over 3700 species are recognised worldwide, with many new species described annually (DeWalt and Ower 2019). Stonefly larvae are fully aquatic and are often dominant food-web components in temperate freshwater ecosystems (Stewart 2009). Stoneflies are sensitive to environmental changes and are often used as model species for biomonitoring studies on freshwater stream health (Zweig and Rabeni 2001). Overall, the sensitivity to local water quality conditions has led to stoneflies being considered the third most imperilled group of aquatic organisms behind only freshwater bivalves and crayfish (Master et al. 2000).

Changes in water temperature can affect life history characteristics of aquatic insect taxa (Sweeney and Vannote 1986). Stonefly distributions in particular may be altered because of future changes in climate (Sheldon 2012). Although many species inhabit perennial streams, several stonefly taxa occupy intermittent systems that experience reduced flow or complete drying during one or more seasons (Bogan and Carlson 2018). This disturbance in flow regime is important for species that are present in karst limestone landscapes. Mississipian-age limestone is permeable and readily eroded by weak carbonic and strong sulfuric acids in rainwater, allowing water to permeate into subterranean levels. The interplay between surface water and subsurface drainage causes channels to become desiccated during periods of drought (White 2002). Intermittent streams still have hyporheic zones under the streambed, thereby providing refugia for macroinvertebrates (Harper 1970).

Surviving the harsh conditions of seasonal intermittent streams requires stoneflies to have well-adapted life history strategies (Woods et al. 2005). Species which are successful in intermittent reaches have univoltine-fast development, where larva either hatch quickly from eggs and migrate downwards to hyporheic regions where water is still available and experience a period of larval diapause or halted egg development until flow resumes (Snellen and Stewart 1979, Harper 1990, Stewart and Anderson 2009, Bogan 2017). Stoneflies, unable to survive in intermittent stream habitats, have univoltine-slow, semivoltine or merovoltine historical strategies where larva grow slowly through the summer or take multiple years to reach maturity (Lechleitner and Kondratieff 1983, Dobrin and Giberson 2003).

Stream permanence may act as a filter for species diversity. For example, stoneflies are restricted to freshwater systems and ecological constraints, such as flow intermittency, may limit regional diversity patterns (Sheldon and Warren Jr 2009). In general, species must pass through multiple levels of environmental filters at hierarchical spatial scales in order to be well established in local communities (Tonn 1990, Poff 1997). At large scales, filters may consist of historical disturbances and climate constraints. Local filters for aquatic insects include watershed characteristics, substrate type and channel size (Poff 1997). These local environmental conditions can be important determinants of local species communities, as well as ultimately acting as strong influences on regional diversity (Huston 1999, Heino et al. 2007). This concept of ecological filtering supports the notion that assemblages must match local conditions in the environment (Janzen 1985).

The overall objective of this study was to assess the influence of stream permanence on stonefly assemblage composition and distribution at two broad landscapes in the Mammoth Cave region, Kentucky, USA. Two main questions were addressed:

1. How different is community assemblage composition between perennial and intermittent streams? This question was addressed mainly by comparing communities of small, upland headwater streams that differ in flow permanence.

2. How do individual species life history characteristics correlate with flow permanence?

## Materials and methods

### Study area

**Figure 1.**
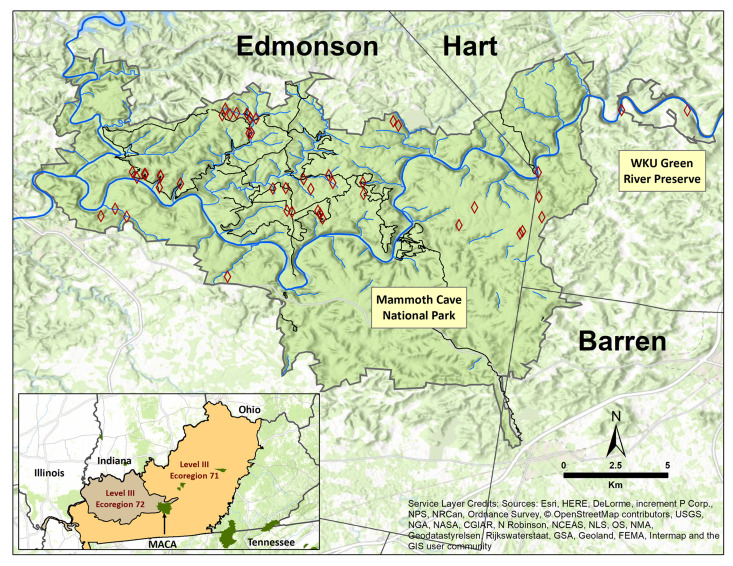
Location of sampling sites within Mammoth Cave National Park (MACA) and the Western Kentucky University Green River Preserve with reference to rivers, streams and backcountry trails. Several spring runs and spring seeps are not mapped. The position of MACA within the Kentucky portions of EPA Level III Ecoregions 71 (Interior Plateau) and 72 (Interior River Valley and Hills Level) is depicted in the lower left inset.

Fieldwork was performed in two near-adjacent landscapes. Mammoth Cave National Park (MACA) is a 21,380 ha (52,830 ac) US national park located primarily in Edmonson County and extending into areas of Hart and Barren Counties of central Kentucky (Fig. 1). The Western Kentucky University (WKU) Green River Preserve (GRP) is 648 ha (1600 ac) and is located 3 km west of MACA in Hart Co. There are portions of two US EPA Level III Ecoregions present at GRP and MACA (Woods et al. 2002; Fig. 1). The Interior Plateau Level III Ecoregion (= 71) is characterised by Mississippian limestone valleys and sandstone cliffs. Subterranean streams, springs, karst windows and very low surface stream density are characteristic of this region and specifically within MACA and GRP. The Interior River Valley and Hills Level III Ecoregion (= 72) at MACA and GRP is mostly Mississippian sandstones and some limestone. The 7^th^ order Green River bisects both GRP and MACA (Fig. 1).

The climate of the region is considered humid mid-temperate with relatively mild winters (National Oceanic and Atmospheric Administration 2012). The average January low temperature is approximately -5°C (23°F) with warm summers having an average July high temperature of 31°C (88°F) (United States 2012). Forest vegetation at MACA is comprised of mostly second-growth forests with very small areas of old growth. Since the Park was mainly farming fields prior to becoming a protected area in 1941, old field sites are dominated by eastern red cedar (*Juniperus virginiana* L.) and Virginia pine (*Pinus virginiana* Mill.) (Olson and Noble 2005). The more mature upland forests are generally oak-hickory (*Quercus velutina* Lam., *Q. alba* L., *Carya cordiformus* (Wangenh.) K. Koch), American beech (*Fagus grandifolia* Ehrh.), maples (*Acer rubrum* L., *A. saccharum* Marshall) and tulip-poplar (*Liriodendron tulipifera* L.) (Olson and Noble 2005). The WKU has a variety of upland habitats, including evergreen stands comprised of Virginia pine, loblolly pine (*P. taeda* L.) and eastern red cedar. Upland and mesic deciduous forests consist of white and black oaks, sugar maple, Ohio buckeye (*Aesculus glabra* Willd.), American beech and shagbark hickory (*C. ovata* (Mill.) K.Koch) (WKU Green River Preserve 2012).

### Field methods

Stonefly adults were collected monthly at 45 sites (Table 1) from December 2018–November 2019. There were 43 MACA sites, including one along the Green River. There were two GRP sites, also including a Green River site. The 45 sites were chosen to represent the broadest range of stream size and flow conditions at MACA and GRP, including some previously-established research locations (Parker 2016). Many sites were accessed through backcountry trails (Fig. 1) and service roads. Each sampling event was time structured for 30 minutes per site to standardise effort. Adults were collected using a beating sheet to dislodge individuals from streamside vegetation and hand-picked off trees, bridges, emergent leaf packs, woody debris and rocks. Since larger-bodied predators are often less abundant, benthic larval collections occurred during December 2019–January 2020 to avoid underestimating representation mainly in the families Perlidae and Perlodidae. Sampling occurred with the use of a standard D-frame net and hand-picking from rocks and leaf packs (Stein et al. 2008). All adult and larval specimens were preserved in 95% undenatured ethanol. All specimen data, accrued during this project, were archived in Darwin Core Archive format supported by Pensoft's Integrated Publishing Toolkit (McRoberts 2021)

**Figure 2.**
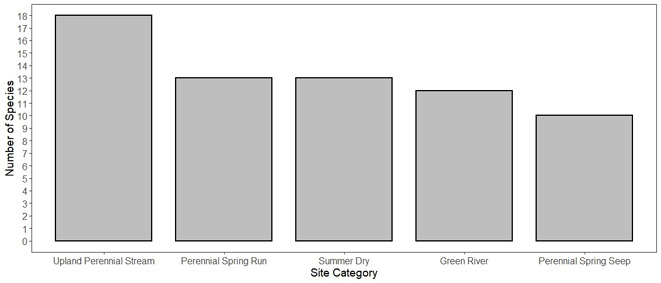
Number of sites in each assigned stream category.

Sites were grouped into five categories, based on channel form and flow permanency (Feminella 1996). These categories consisted of perennial spring runs (springheads with a defined stream channel, typically at the source), perennial spring seeps (springheads lacking a defined stream channel, typically at the source), upland perennial streams, summer dry streams and the Green River. In total, 71% of sites were a combination of perennial spring runs (17) and summer dry streams (15). This was followed by six upland perennial streams, five perennial spring seeps and two along the Green River (Fig. 2). GPS coordinates for each site were taken with a Garmin GPSMAP 64st GPS unit and subsequently georeferenced for accuracy using ACME Mapper 2.2. Drainage area of each site was calculated using the watershed feature in ArcMap 10.7. Wetted width and dominant substrate composition were recorded during benthic sampling events in December 2019.

### Statistical methods

The freeware programme EstimateS version 9.1.0 calculated projected species richness of the region by inputting presence-only data for species found during each monthly sampling event (Colwell 2006). The EstimateS output also produced a graphical representation of species rarity in the form of singletons and doubletons. Hierarchical clustering was done using an unweighted pair group method with arithmetic mean (UPGMA) comparison of community composition amongst the five stream categories. The UPGMA and all bar graphs created for data visualisation were accomplished using the freeware programme R 1.2.5001 (R Core Team 2013).

Life history strategies for all species reported in this study were categorised as univoltine-fast, univoltine-slow, semivoltine and merovoltine. As life history studies have not occurred for all species found in this study (e.g. *Allocapnia mystica* Frison, 1929), specific strategies were inferred from literature using species in the same genus (Stewart and Stark 2002).

## Results

### Assemblage composition

**Figure 3.**
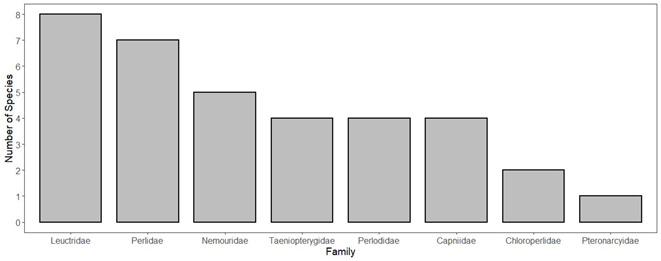
Number of species collected from each family.

In total, 1562 adults and 36 larvae were collected comprising 34 recognised species. This represented eight families (Fig. 3). Leuctridae was the most speciose family (n = 8), followed by Perlidae (n = 6), Nemouridae (n = 5) and four each in Capniidae, Perlodidae and Taeniopterygidae. The five most common species were *Allocapnia recta* (Claassen, 1924) (22 unique localities and 27% of all individuals collected), *A. rickeri* Frison, 1942 (12 localities, 10%), *Amphinemura varshava* (Ricker, 1952) (22 localities, 6%), *A. nigritta* (Provancher, 1876) (20 localities, 7%) and *Leuctra schusteri* Grubbs, 2015 (18 localities, 10%) (Table 2).

**Figure 4.**
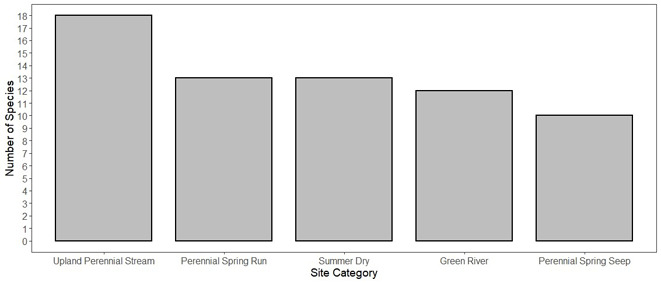
Number of species collected from each stream category.

**Figure 5.**
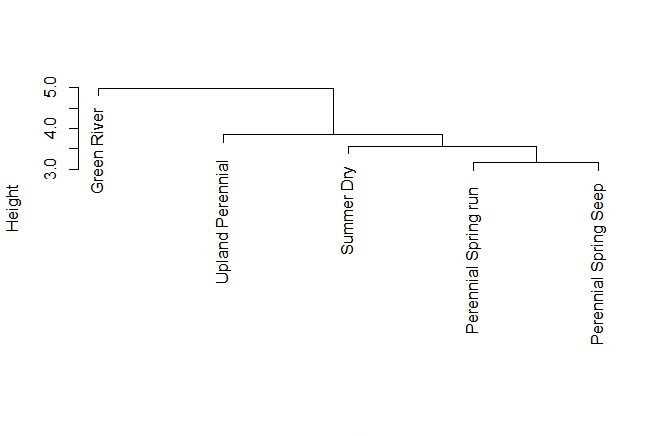
UPGMA analysis showing species similarity between species found at each stream category.

With one exception, species richness was similar across each stream category. The largest number of species collected were from upland perennial streams (n = 17), followed by 13 species each from summer dry streams and perennial spring runs, 11 from the Green River and 10 from perennial spring seeps (Fig. 4). The stonefly communities found in perennial spring runs and perennial spring seeps were the most similar to each other (Fig. 5) and reflected overall similarities in life history strategies (Table 3). The summer-dry category consisted only of species exhibiting univoltine-fast life cycles. This category also had species found in both the perennial spring runs and perennial spring seeps. The upland perennial stream and Green River communities had more species that were merovoltine or did not undergo a period of diapause. The summer-dry streams were most different from the upland perennial stream and the Green River assemblages (Fig. 5).

Species emergence was successional as expected, with adults present in all months (Table 4). Adult presence varied across species, as some species had extended emergence periods while others were only collected within very short timeframes (e.g. once from one stream). Distribution of species across MACA and GRP also varied. Species collected in each stream category are documented individually below, with notes on adult presence and distribution. Family names were arranged in phylogenetic order.

#### Family Capniidae

*Allocapnia granulata* (Claassen, 1924). This species was only collected from the two Green River locations (Fig. 6a). Adult presence was from mid-January through late March (Table 4). This is a widespread species ranging from north-eastern Canada southwards to the southern United States and west to Oklahoma and Kansas (DeWalt et al. 2020).

*Allocapnia mystica* Frison, 1929. This species was found primarily in summer dry sites with one additional record from an upland perennial stream. These sites were north of the Green River and most were clustered in a small area in the northwest section of MACA (Fig. 6b). Adults were only collected in early February (Table 4). This is a common species in unglaciated areas of the United States (DeWalt et al. 2020).

*Allocapnia recta* (Claassen, 1924). This species was collected from all five stream categories, but was most abundant from summer dry and upland perennial streams (Fig. 6c). Adult presence lasted from mid–late October through March (Table 4). This species was widespread through MACA, but not collected at GRP. *Allocapnia recta* is widespread and distributed throughout most of the eastern United States and eastern Canada (DeWalt et al. 2020). Grubbs et al. (2006) reported a univoltine-slow life cycle for this species from both a spring run and a small intermittent stream in the Mammoth Cave region.

*Allocapnia rickeri* Frison, 1942. This species was collected primarily from perennial spring runs and summer dry sites at MACA, but also from upland perennial streams and spring seeps (Fig. 6d). Adults were present from early December through late March (Table 4). This is a common and widely-distributed species throughout eastern North America (DeWalt et al. 2020).

#### Family Leuctridae

*Leuctra alta* James, 1974. This species was very abundant in MACA, mainly in summer dry streams (Fig. 7a). Adults were collected from late March through May (Table 4). *Leuctra alta* is a mid-western and southern US Appalachian species (Grubbs 2005, Grubbs and Sheldon 2018, DeWalt et al. 2020). Grubbs et al. (2006) reported a univoltine-fast life cycle for a *L. alta*-*L. sibleyi* mix from both a spring run and a small intermittent stream in the Mammoth Cave region.

*Leuctra rickeri* James, 1976. This species was collected across MACA and found in all stream categories, except the Green River (Fig. 7b). Adults were present from early May until late June (Table 4). This species occurs from north-western Florida north to Iowa, Michigan and Maryland (DeWalt et al. 2020).

*Leuctra schusteri* Grubbs, 2015. This is a common and an apparently endemic species to the Mammoth Cave region (Grubbs 2015) and was abundant in collections from perennial spring runs and perennial spring seeps (Fig. 7c). Adults were present for an extended period of the year from early July through late January (Table 4). Grubbs et al. (2006) reported a univoltine-slow life cycle for this species under the name L. cf. tenuis from a spring run in the Mammoth Cave region.

*Leuctra sibleyi* Claassen, 1923. This species was found across MACA from all stream categories, except the Green River (Fig. 7d). Adult presence mirrored that of *L. alta* and lasted from late March through May (Table 4). This species is broadly distributed across North America east of the Mississippi River (DeWalt et al. 2020). Grubbs et al. (2006) reported a univoltine-fast life cycle for a *L. alta*-*L. sibleyi* mix from both a spring run and a small intermittent stream in the Mammoth Cave region.

*Leuctra tenuis* (Pictet, 1841). This species was only present in upland perennial streams in the northwest portion of MACA (Fig. 8a). Adult presence lasted from early August through mid-October (Table 4). The range of this species includes most of the eastern United States and eastern Canada (Grubbs 2015, DeWalt et al. 2020).

*Paraleuctra sara* (Claassen, 1937). This species was collected as adults only from three sites, two upland perennial streams and one perennial spring seep, all from north of the Green River (Fig. 8b). Adult presence lasted from mid-March through May (Table 4). *Paraleuctra sara* is distributed widely in the eastern United States, westwards to Oklahoma and Arkansas and north to southern Canada (Stark and Kyzar 2001, DeWalt et al. 2020).

*Zealeuctra claasseni* (Frison, 1929). This species was only collected as adults from two summer dry streams at MACA in April (Fig. 8c, Table 4). This species occupies most of the unglaciated southern United States, from Texas north to Kansas and the lower Midwest and northeast to West Virginia (Grubbs et al. 2013, DeWalt et al. 2020).

*Zealeuctra fraxina* Ricker & Ross, 1969. This species was collected as adults from three sites, one each from summer-dry, perennial spring run and an upland perennial stream in the northwest corner of MACA (Fig. 8d). Adults were collected from late-January through March (Table 4). This species occupies unglaciated landscapes east of the Mississippi River north to New Jersey and Pennsylvania (Grubbs et al. 2013, DeWalt et al. 2020).

#### Family Nemouridae

*Amphinemura alabama* Baumann, 1996. This species was collected as adults commonly throughout MACA and was found in every stream category, except for the Green River (Fig. 9a). Adults were collected from mid-March through April (Table 4). This species ranges from the northern tiers of Alabama and Mississippi northwards to central Kentucky (Stark & Harrison 2010, DeWalt et al. 2020).

*Amphinemura nigritta* (Provancher, 1876). This species was very common across MACA and was collected as adults in all stream categories, except for the Green River (Fig. 9b). This species was collected most abundantly from summer dry sites. Adult presence lasted from early March to June (Table 4). *Amphinemura nigritta* is distributed extensively across eastern North America (DeWalt et al. 2020).

*Amphinemura varshava* (Ricker, 1952). Identical to *A. nigritta*, this species inhabits the same four stream categories, but was found more abundantly in the northern half of MACA (Fig. 9c). This was the only species collected from the GRP spring seep. Adult presence was also similar to *A. nigritta* and lasted from early March to June (Table 4). This species has a range across most of the mid-western USA, from Iowa east to Illinois and southwest to Georgia (DeWalt et al. 2020). Grubbs et al. (2005) reported a univoltine-fast life cycle for this species from a small intermittent stream in the Mammoth Cave region.

*Ostrocerca truncata* (Claassen, 1923). This species was collected as adults from perennial spring runs, perennial spring seeps and summer dry streams (Fig. 9d). Adult were collected in April and May (Table 4). This is a common Appalachian species with westward distributions to central Indiana and central Kentucky (Young et al. 1989, DeWalt et al. 2020). Grubbs et al. (2005) reported a univoltine-fast life cycle for this species from an intermittent stream in the Mammoth Cave region.

*Soyedina calcarea* Grubbs, 2006. This is a very common and abundant species at MACA, collected from perennial spring seeps and perennial spring runs (Fig. 10a). Adults were present from late January through mid-April (Table 4). This species appears to be endemic to the Mammoth Cave region (Grubbs and Baumann 2019). Grubbs (2006) reported a univoltine-slow life cycle for this species from a spring run in the Mammoth Cave region.

#### Family Taeniopterygidae

*Strophopteryx fasciata* (Burmeister, 1839). This species was collected only once in late January at the MACA Green River site (Fig. 10b, Table 4). *Strophopteryx fasciata* has a broad range across most of eastern North America (Stewart 2000, DeWalt et al. 2020).

*Taeniopteryx burksi* Ricker & Ross, 1968. This species was collected from both Green River sites (Fig. 10c) and also from one perennial spring seep. Adults were present from December through January (Table 4). *Taeniopteryx burksi* has a broad range across most of central and eastern North America (Stewart 2000, DeWalt et al. 2020).

*Taeniopteryx lita* Frison, 1942. This species was collected from one location only at the GRP Green River site (Fig. 10d). *Taeniopteryx lita* was collected as adults from early December through January (Table 4). This species is common in unglaciated landscapes, distributed from Florida west to Texas and north to Indiana, Ohio and New Jersey (Stewart 2000, DeWalt et al. 2020).

*Taeniopteryx maura* (Pictet, 1841). This species was commonly collected from the GRP and MACA Green River sites (Fig. 11a). Adults were collected in December and January (Table 4). *Taeniopteryx maura* occupies the south-eastern United States north to Maine (Stewart 2000, DeWalt et al. 2020).

#### Family Pteronarcyidae

*Pteronarcys dorsata* (Say, 1823). This species was not collected as an adult, but was identified from both Green River sites from exuviae (Fig. 11b; Myers and Kondratieff 2018). Exuviae were found in early April and mid-June, which indicates adult presence during the same time of year (Table 4). *Pteronarcys dorsata* is widely reported from across much of North America (Nelson 2000, DeWalt et al. 2020).

#### Family Chloroperlidae

*Haploperla brevis* (Banks, 1895). This species was collected from only one upland perennial stream in late May (Fig. 11c, Table 4). This was surprising since *H. brevis* is a common species with a range encompassing most of the eastern United States and southern Canada (Surdick 2004, DeWalt et al. 2020). The gap in collecting dates between early April and late May might be responsible for a spuriously low number of records for MACA.

*Sweltsa hoffmani* Kondratieff & Kirchner, 2009. This species was found from two sites north of the Green River, once each from perennial seep spring and a perennial spring run (Fig. 11d) in early April and late May (Table 4). *Sweltsa hoffmani* species is distributed from Indiana east to the southern Appalachian Mountain region (Surdick 2004, Kondratieff and Kirchner 2009, Grubbs 2011a, DeWalt et al. 2020).

#### Family Perlidae

*Acroneuria abnormis* (Newman, 1838). This species was abundant as adults and larvae and were found in upland perennial streams and perennial spring runs (Fig. 12a). Adult presence spanned from early May through mid-June (Table 4). *Acroneuria abnormis* is distributed extensively across North America (Stark 2004, DeWalt et al. 2020).

*Acroneuria perplexa* Frison, 1937. One female was collected from the MACA Green River site in late May (Fig. 12b, Table 4). This species is most commonly reported from rivers from Oklahoma east to Georgia and north to Pennsylvania (Stark 2004, DeWalt et al. 2020).

*Eccoptura xanthenes* (Newman, 1838). This distinctive species was collected as an adult in early June and as larvae from two MACA spring seeps (Fig. 12c, Table 4). *Eccoptura xanthenes* is distributed mainly from Florida north to New York (Stark 2004, DeWalt et al. 2020).

*Neoperla stewarti* Stark & Baumann, 1978. One male was collected from the MACA Green River site in early June (Fig. 12d, Table 4). This species occurs across much of eastern North America (Stark 2004, DeWalt et al. 2020).

*Perlinella drymo* (Newman, 1839). This species was collected only from the GRP Green River site in early June (Fig. 13a, Table 4). *Perlinella drymo* ranges from Texas across eastern North America and northwards to Minnesota and areas of Canada (Stark 2004, DeWalt et al. 2020).

*Perlinella ephyre* (Newman, 1839). This species was also collected from the same GRP Green River site as *P. drymo* in early June (Fig. 13b, Table 4). *Perlinella ephyre* has a range similar to *P. drymo* (Stark 2004, DeWalt et al. 2020).

#### Family Perlodidae

*Clioperla clio* (Newman, 1839). This species was commonly found across MACA primarily from perennial spring runs and upland perennial streams (Fig. 13c). Adult presence spanned from early April through May (Table 4). This species is distributed widely across eastern North America (Szczytko and Kondratieff 2015, DeWalt et al. 2020).

*Diploperla robusta* Stark & Gaufin, 1974. This species was found as adults less commonly than *C. clio*, once each from a perennial spring run, an upland perennial stream and a summer dry stream (Fig. 13d). Adults were all collected in May (Table 4). *Diploperla robusta* occurs from Alabama north to Illinois and northeast to Connecticut (Kondratieff 2004, DeWalt et al. 2020).

*Isoperla kirchneri* Szczytko & Kondratieff, 2015. This species was found in abundance from three upland perennial streams (Fig. 14a). Adult presence was from early April through late June (Table 4). This species is known from Kentucky and Tennessee northeastwards to Pennsylvania and New York (Szczytko and Kondratieff 2015, DeWalt et al. 2020).

*Isoperla powhatan* Szczytko & Kondratieff, 2015. This species was only collected from one upland perennial stream near the middle of MACA in late May (Fig. 14b, Table 4). This is a new state record and the westernmost-known locality of this species, with prior records from North Carolina, Pennsylvania and Virginia (Szczytko and Kondratieff 2015, DeWalt et al. 2020).

### Influence of stream permanence on species richness patterns

**Figure 15.**
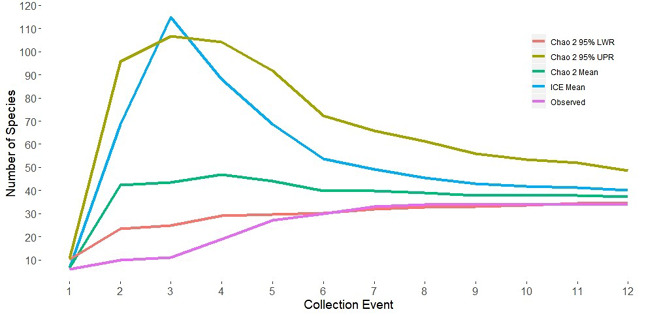
Estimations of species richness values.

**Figure 16.**
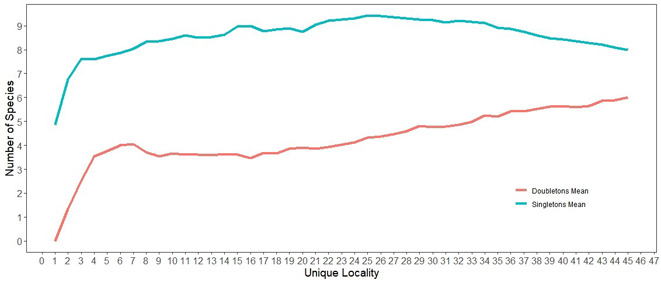
Estimations of species rarity as singletons and doubletons.

Although 34 species were collected, species richness indicators predicted higher values. The Choa2 estimator had a mean of 37.2, with the 95% confidence intervals ranging from 35 to 49 and the ICE mean value was 40.1 (Fig. 15). There were 14 species found at only one or two sites as eight singletons and six doubletons (Fig. 16). Most of these 14 species have presumed univoltine-slow or merovoltine life cycles, indicating stream permanence as a limiting factor in habitat availability. Moreover, 11 species were only collected from the Green River. An artifact of the sampling design was that only two localities along the Green River were included. Hence, a species collected at one or both locations along the Green River (and perhaps a common regional species found in small rivers such as *A. granulata*) can only be a singleton or doubleton.

### Distributions of species life history traits

Most species (n = 30) collected exhibit or likely exhibit univoltine life cycles, while the other four have multiyear cycles (Table 3). Species with univoltine-fast life cycles, where nymphal growth is completed rapidly within a few months, were also collected more commonly than species with univoltine-slow life cycles. Species with egg or nymphal diapause were also found commonly in the region vs. species lacking this adaptation for channel desiccation (Table 3). Overall, the niche traits of species found most commonly appear to reflect small upland stream sites that are either summer-dry or summer-wet. For species collected that exhibit univoltine-fast life cycles and found in intermittent reaches (e.g. *L. alta*), adult emergence and reproduction was completed by late summer.

## Discussion

This study addressed two related questions pertaining to the stonefly community present mainly at Mammoth Cave National Park, USA. The well-developed carbonate karst landscape underlying MACA manifests itself with numerous springs with perennial flow and several small upland streams that experience full channel drying during summer and autumn (Woods et al. 2005, White 2002). Stonefly community structure overall appeared to be directly reflective of stream size and annual flow patterns.

Aquatic macroinvertebrate communities vary with stream size (Minshall et al. 1985, Grubaugh et al. 1996, Vinson and Hawkins 1998, Paavola et al. 2003, Al-Shami et al. 2013, García-Roger et al. 2013). The two Green River sites were most different from the other four stream categories. With the exception of the Green River, there are only three streams at MACA with wetted widths > 5.0 m during summer low flow conditions. This may restrict habitat availability for other stonefly species inhabiting the region. Species richness estimators indicate several additional species could be found. This could be attributed to at least three factors. One, the most common stream size at GRP and MACA was 1‒3 m wide. This means that species with slow annual development and multiyear life cycles (e.g. *P. dorsata*) and commonly found in mid-order channels or rivers would be less common or absent due to habitat limitations. Two, there was a gap in sampling between early April and late May 2019. There are common regional species that are found in small streams and emerge during spring (e.g. *Alloperla caudata* Frison, 1934 and *Isoperla decepta* Frison, 1935) yet were not collected. Three, the perlid genus *Perlesta* Banks, 1906 was surprisingly not collected during this study. Several species of *Perlesta* are found commonly across central Kentucky, including those found in both small upland streams and small rivers (Grubbs and DeWalt 2008, Grubbs and DeWalt 2012, Grubbs and DeWalt 2018). Notable and perhaps false absences include *P. adena* Stark, 1989, *P. armitagei* Grubbs & DeWalt, 2018 and *P. ephelida* Grubbs & DeWalt, 2012.

Stream macroinvertebrate community structure and function are reflective of annual flow patterns, especially in headwater tributary systems (Feminella 1996, Arscot et al. 2010, Clarke et al. 2010, García-Roger et al. 2011, Grubbs 2011b, Bogan et al. 2013, Datry et al. 2013, Datry et al. 2014, Stubbington et al. 2017). Community richness is typically lower in intermittent streams and community composition is often a smaller subset of taxa found in perennial systems (Vinson and Hawkins 1998, Arscot et al. 2010, Datry et al. 2014). Intermittent streams are a category of temporary systems with adequate groundwater to maintain surface flow during wetter seasons, yet experience prolonged periods of ceased flow during a dry season (Gordon et al. 1992, Datry et al. 2017), Temporary streams, in general, are common in both arid and mesic landscapes, comprising at least 30% of stream drainage networks globally (Tooth 2000, Larned et al. 2010). This absence of water creates a disturbance (Townsend 1989, Lake 2003) and species must rely on life history adaptations to survive (Brittain 1991). Adaptations include short voltinism periods, behavioural capacity to disperse vertically (i.e. into hyporheic flow) or longitudinally (i.e. drift) and desiccation resistance (Williams 1996, Rosario and Resh 2000, Beche et al. 2005, García-Roger et al. 2013).

Perennial spring runs are common at MACA and were representative of the highest proportion of stream types in this study. Stonefly communities were most similar between perennial spring runs and perennial spring seeps. This was not surprising since these two karst habitat types have annual flow permanence and likely have similar daily and annual thermal regimes. The upland perennial streams and the Green River categories included the largest lotic systems sampled and provided annual flow to support species with univoltine-slow and merovoltine life history strategies. This is evidence of stream permanence acting as an environmental filter on communities of stoneflies in the region (Poff 1997). Stream reaches that experience flow intermittency and extended periods of channel drying are also common. Intermittent stream communities across the region partially resemble those of perennial systems, yet lack species unable to survive stream desiccation. Some species, however, were primarily found in intermittent streams (e.g. *L. alta*, *Z. claasseni*). Communities collected in summer dry streams contained only species with univoltine-fast life histories, since species lacking diapause mechanisms would not survive stream desiccation in late summer (Williams 1996).

At the landscape spatial scales across MACA and GRP, community composition is acted upon by local and regional abiotic factors (Poff 1997). An important abiotic factor and a major factor effecting stream permanence is the presence of limestone bedrock across the karst region of central Kentucky (White et al. 1970). Presumed life histories of the stonefly species collected directly reflect the high number of sites with intermittent flow regimes. Out of the 34 total species collected, 30 (88%) are presumed to exhibit univoltine development. Twenty-four species (71%) collected are presumed to have a period of diapause in their life cycle (either in larval or egg form), develop quickly as larvae and reach maturity in one calendar year (Table 3). Species with univoltine-fast voltinism appear better adapted to regional environmental conditions compared to those with slow annual development or multiple year life histories. Hence, most species that do not have univoltine-fast life histories are fundamentally “filtered” out of the region, except for those species that are adapted to the flow permanence of the Green River (Lytle and Poff 2004).

## Conclusions

In summary, the diversity of the stonefly fauna at Mammoth Cave National Park and the WKU Green River Preserve is greatly affected by annual stream flow patterns, which in turn, is a product of living in an environment with underlying limestone bedrock. Species able to survive through the harsh conditions of complete seasonal channel drying do so by life history adaptations, allowing them to access refugia in groundwater or have mechanisms to survive channel desiccation.

## Figures and Tables

**Figure 6a.**
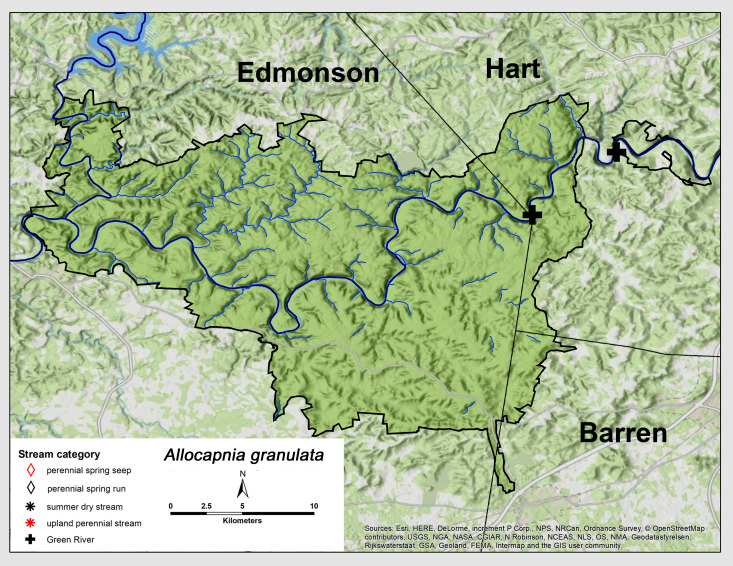
*Allocapnia granulata*

**Figure 6b.**
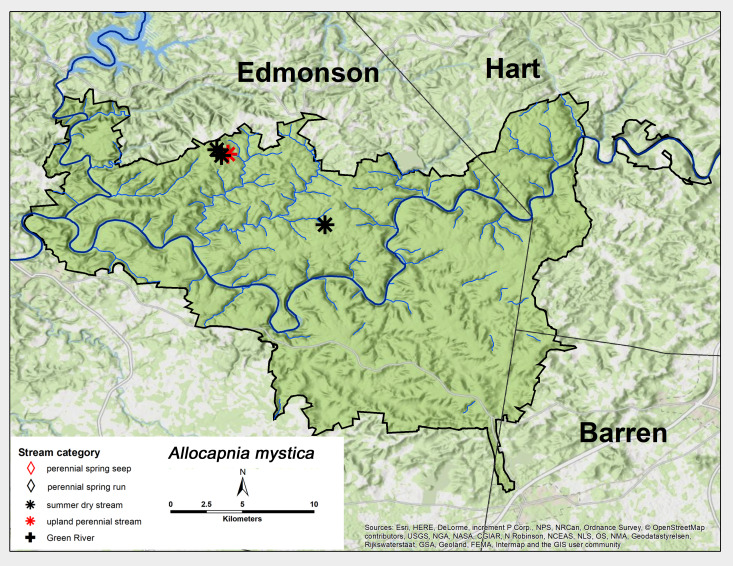
*Allocapnia mystica*

**Figure 6c.**
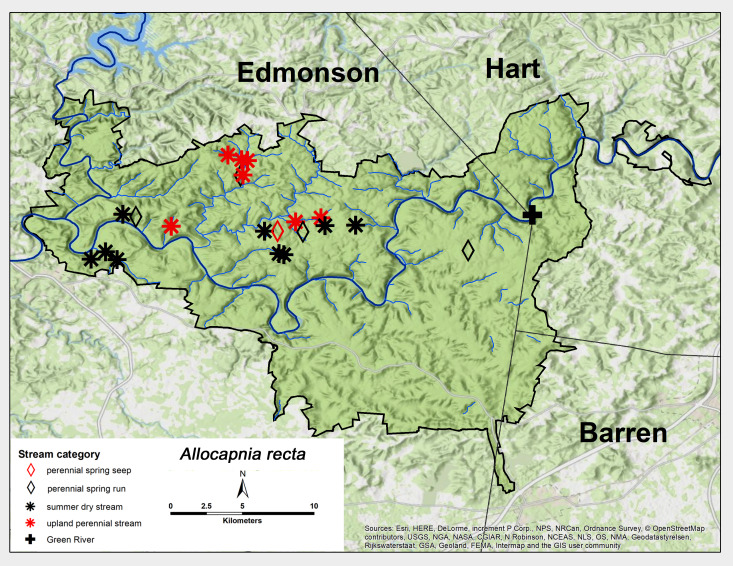
*Allocapnia recta*

**Figure 6d.**
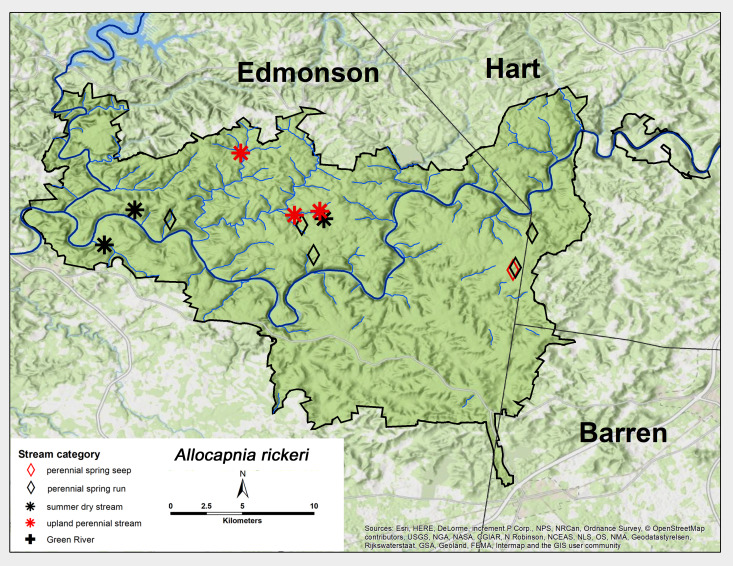
*Allocapnia rickeri*

**Figure 7a.**
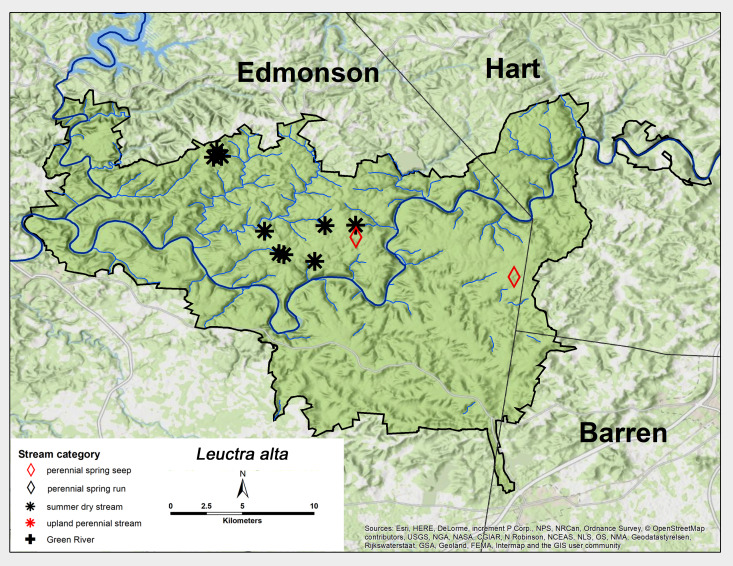


**Figure 7b.**
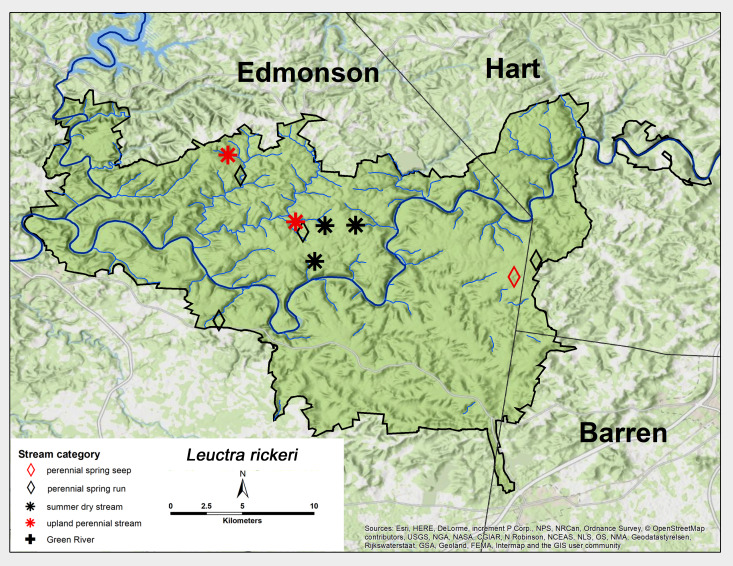


**Figure 7c.**
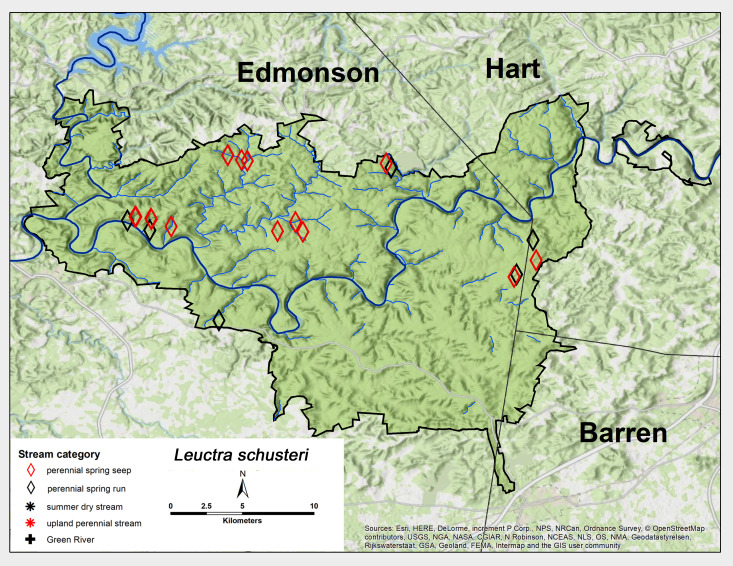


**Figure 7d.**
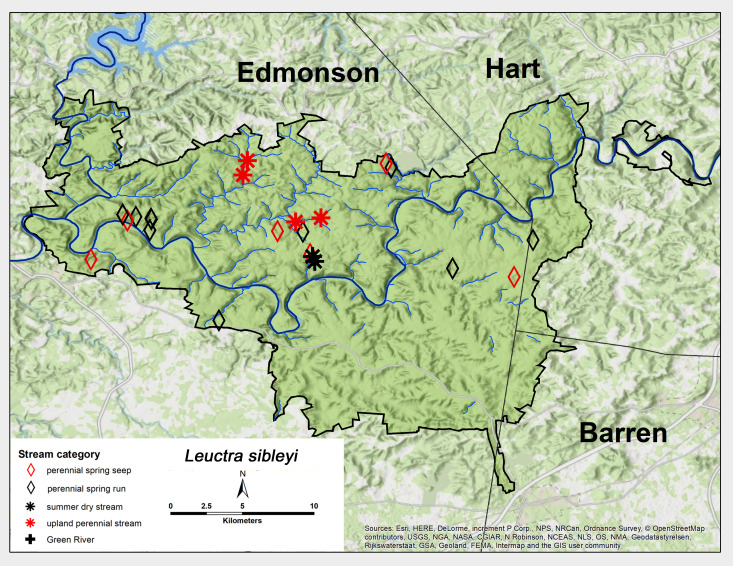


**Figure 8a.**
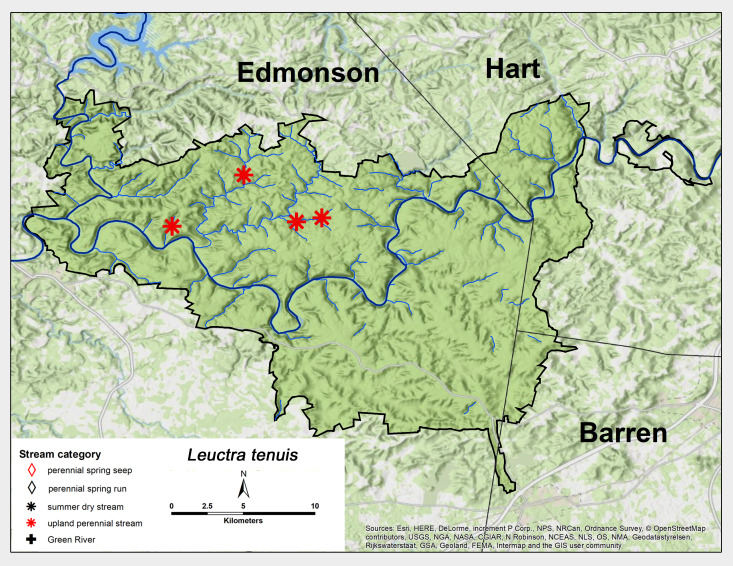


**Figure 8b.**
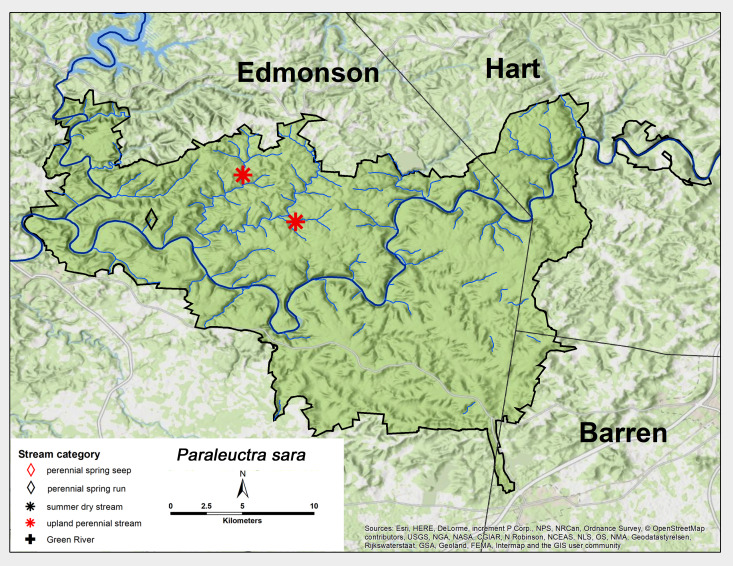


**Figure 8c.**
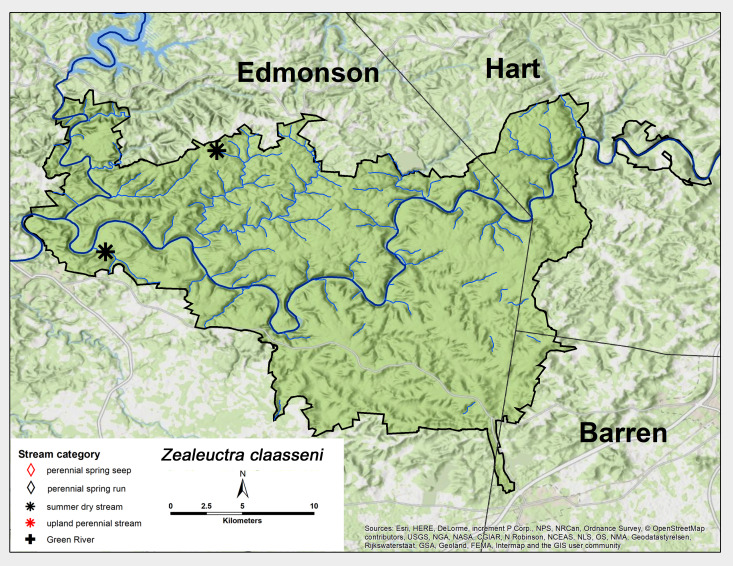


**Figure 8d.**
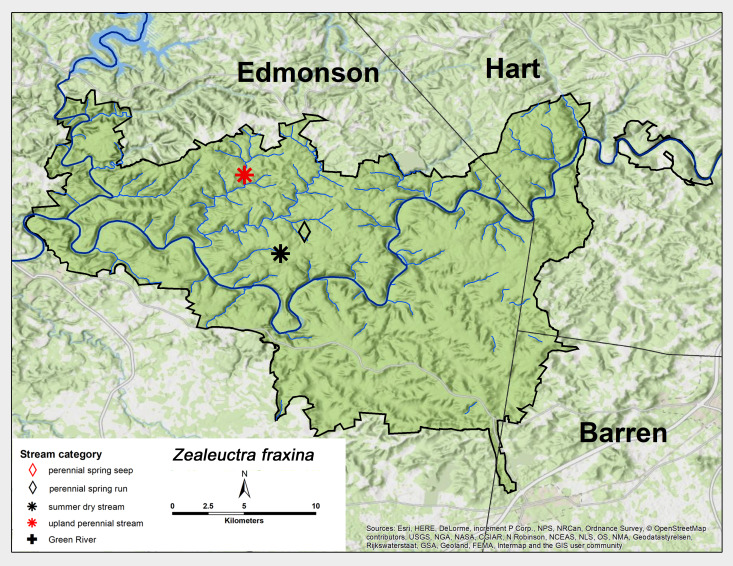


**Figure 9a.**
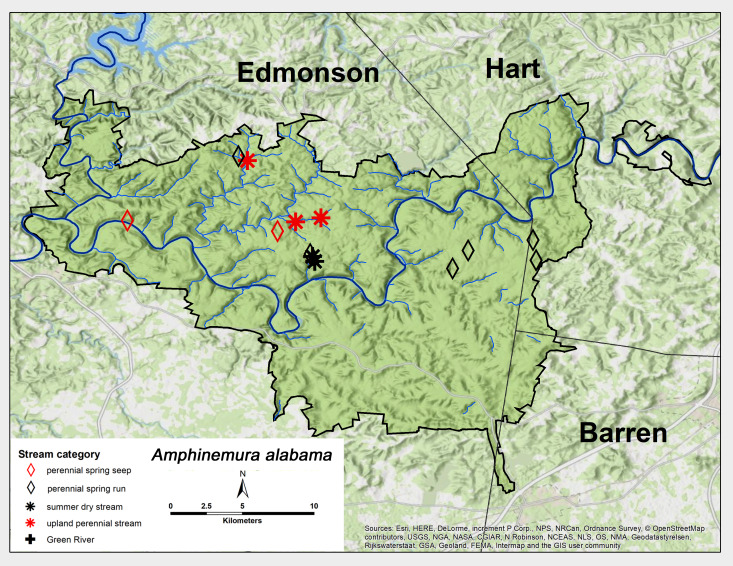


**Figure 9b.**
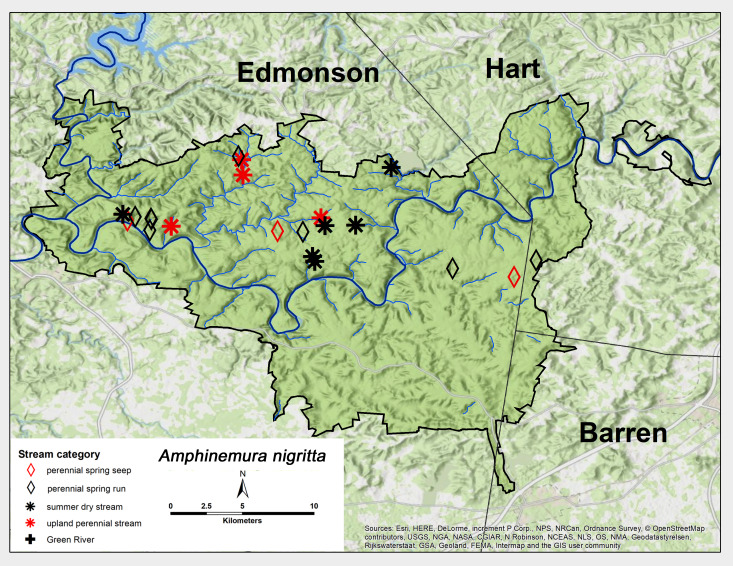


**Figure 9c.**
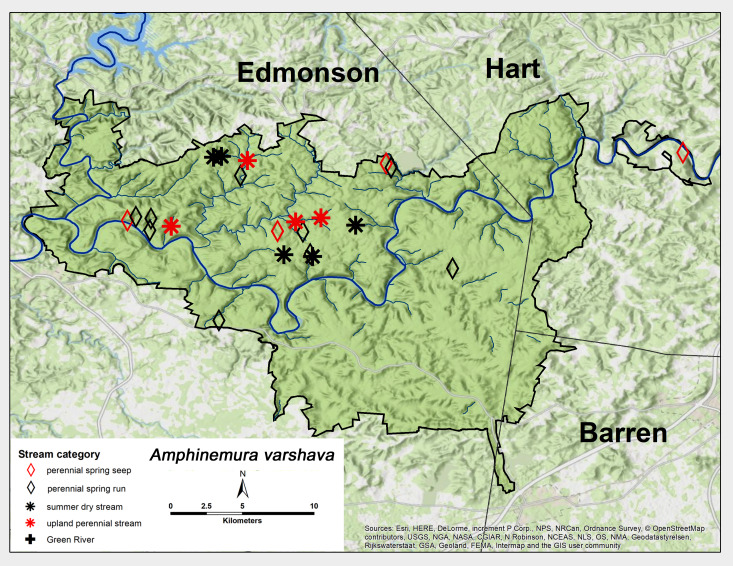


**Figure 9d.**
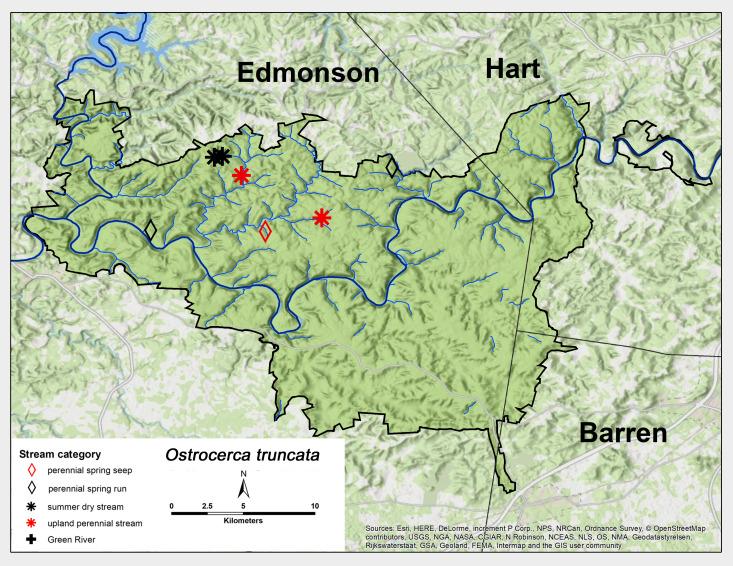


**Figure 10a.**
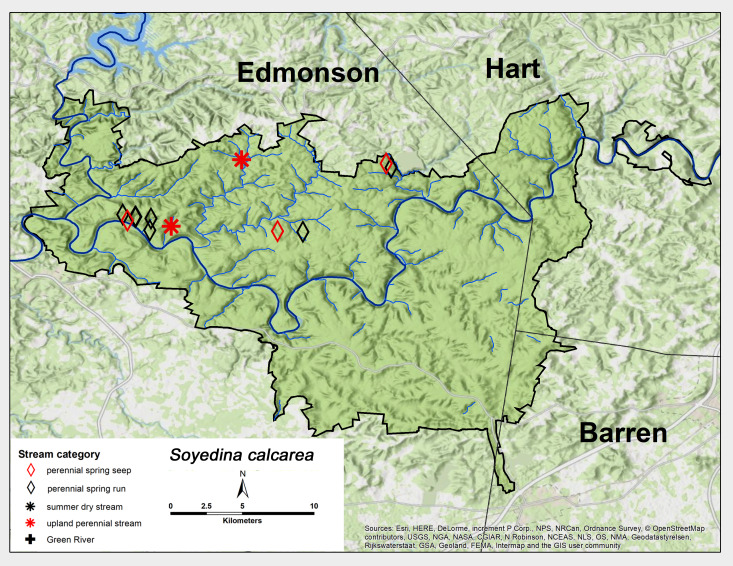


**Figure 10b.**
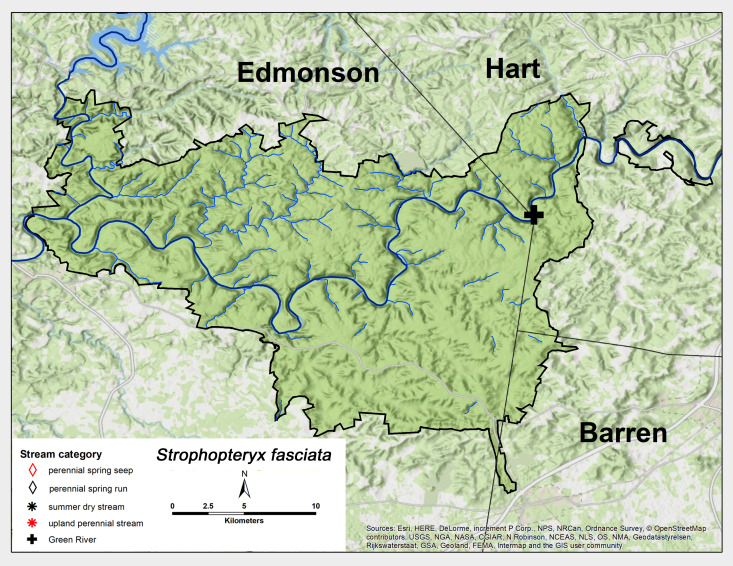


**Figure 10c.**
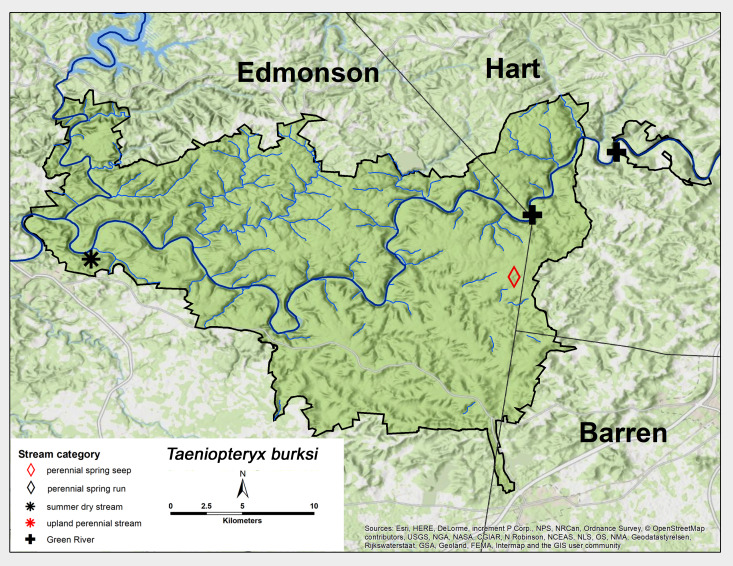


**Figure 10d.**
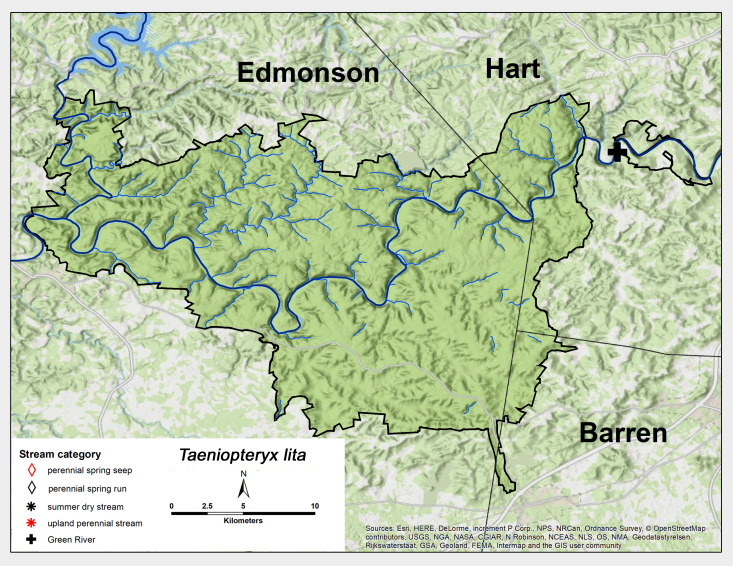


**Figure 11a.**
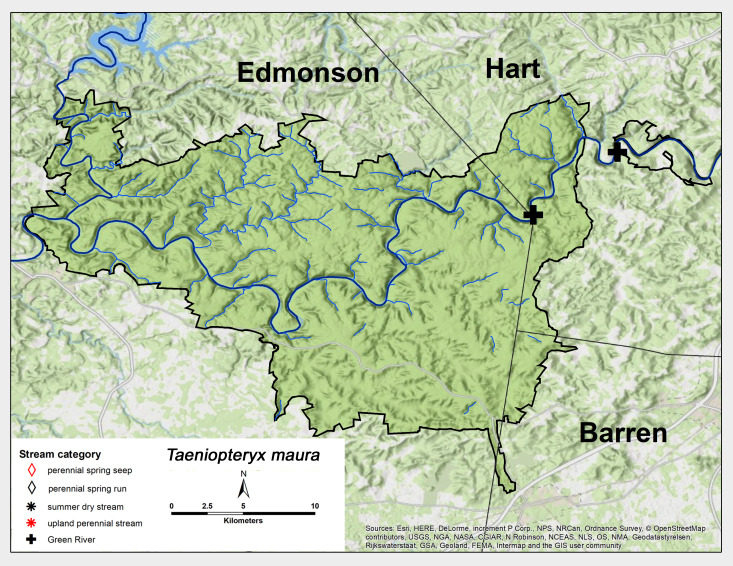


**Figure 11b.**
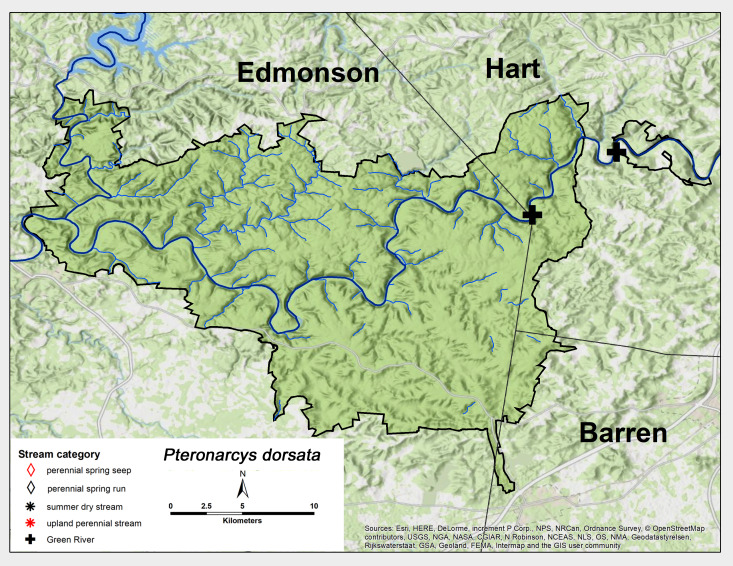


**Figure 11c.**
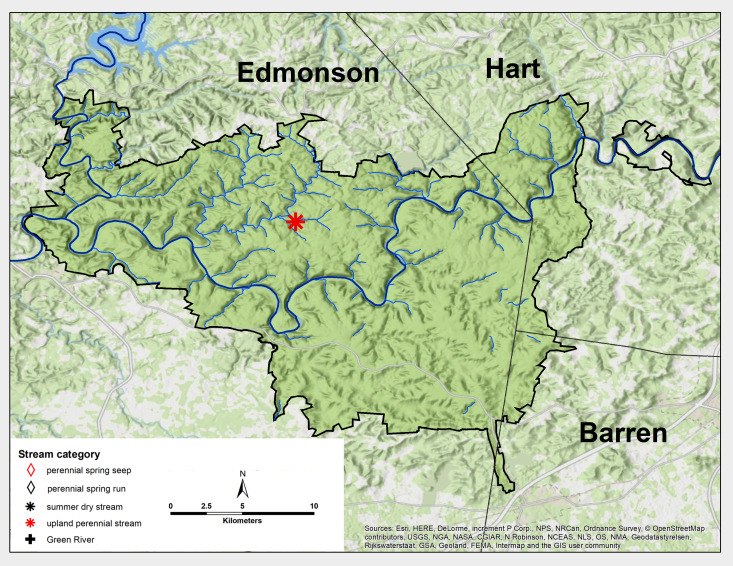


**Figure 11d.**
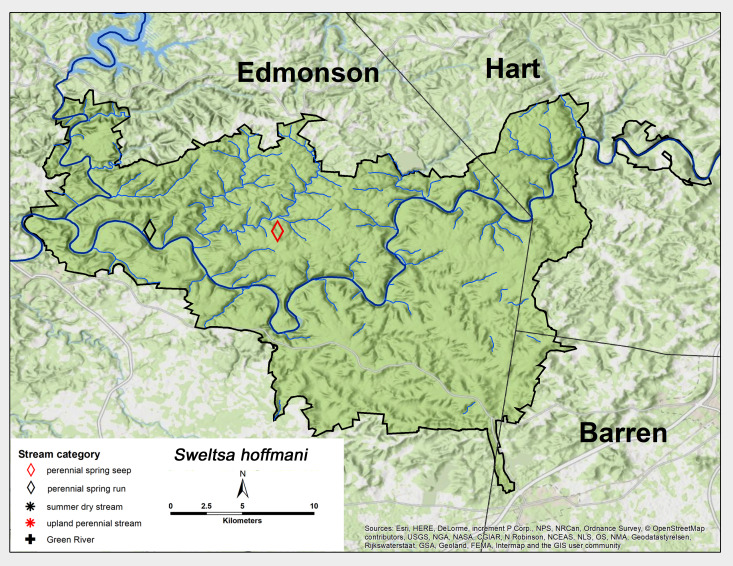


**Figure 12a.**
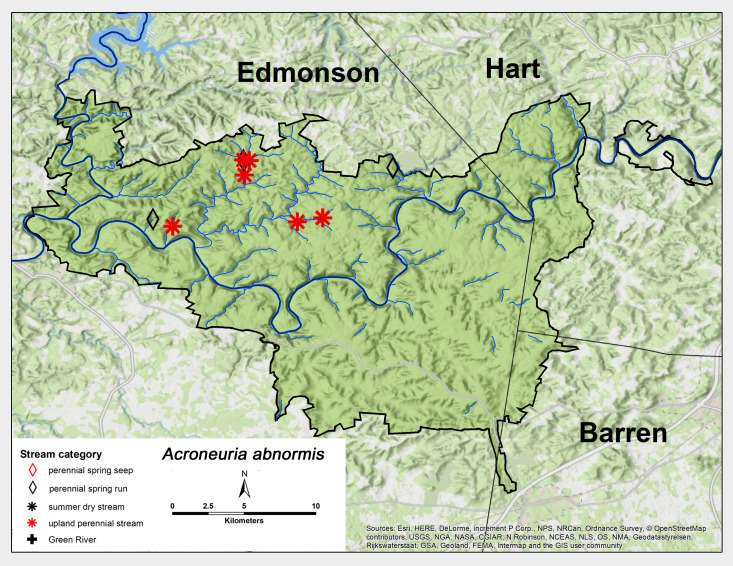


**Figure 12b.**
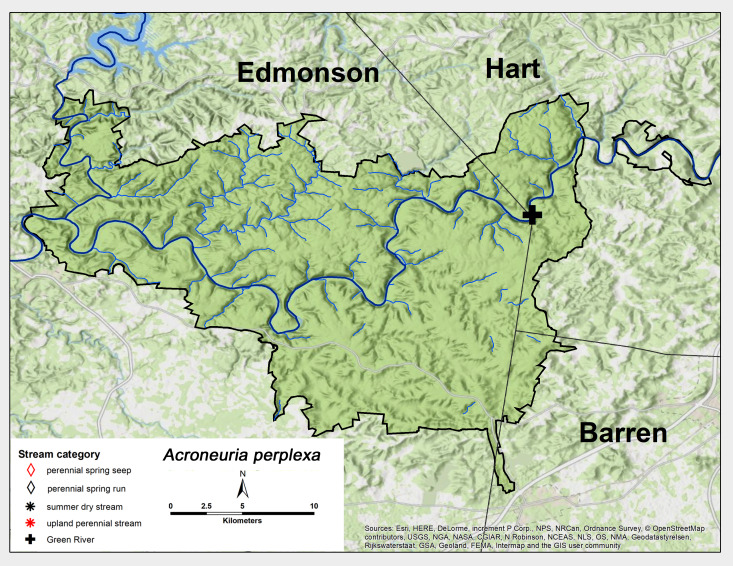


**Figure 12c.**
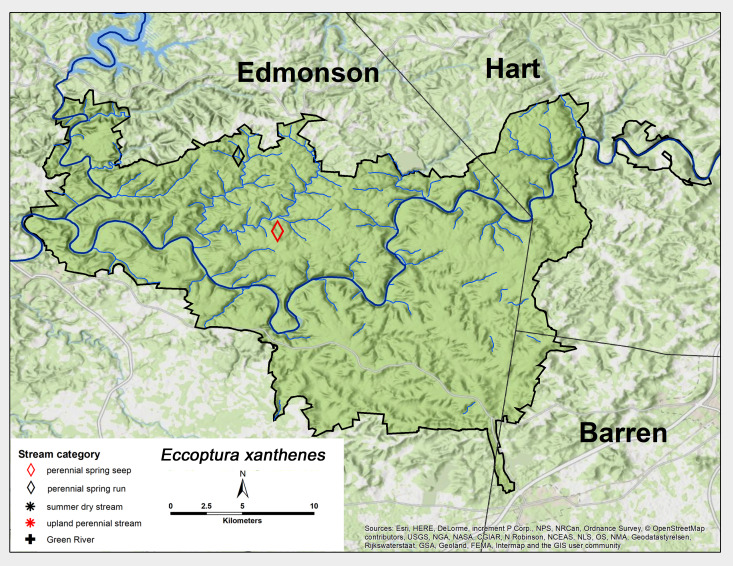


**Figure 12d.**
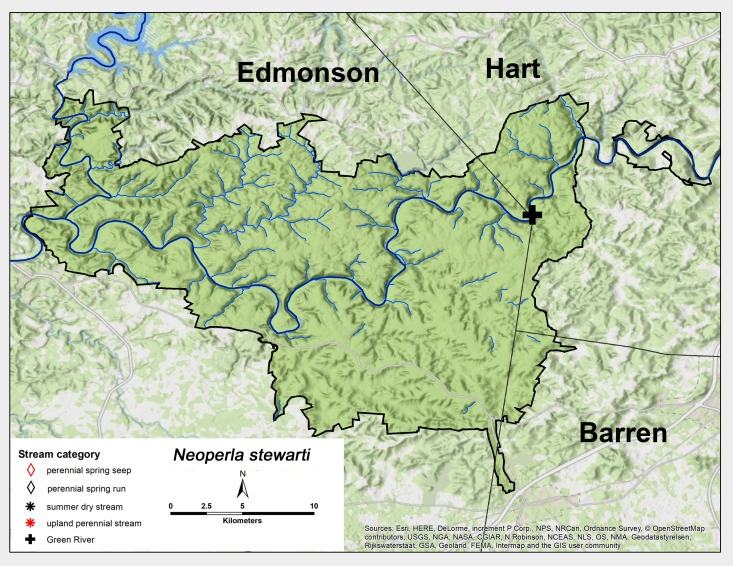


**Figure 13a.**
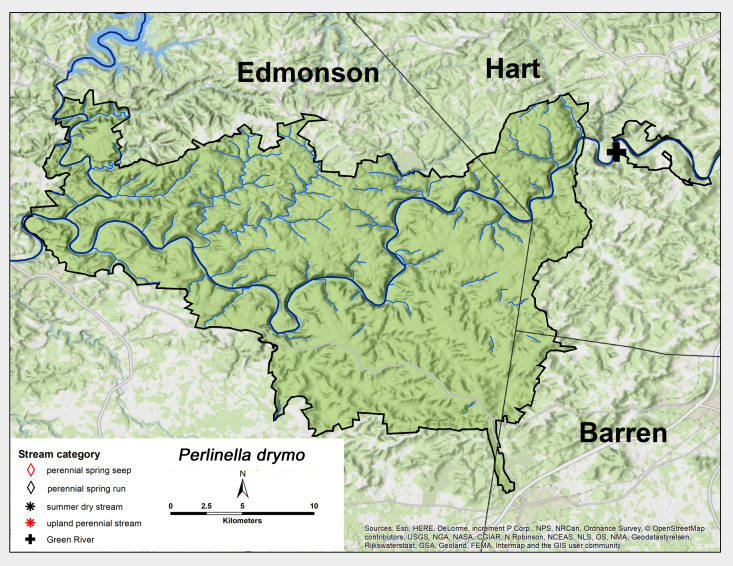


**Figure 13b.**
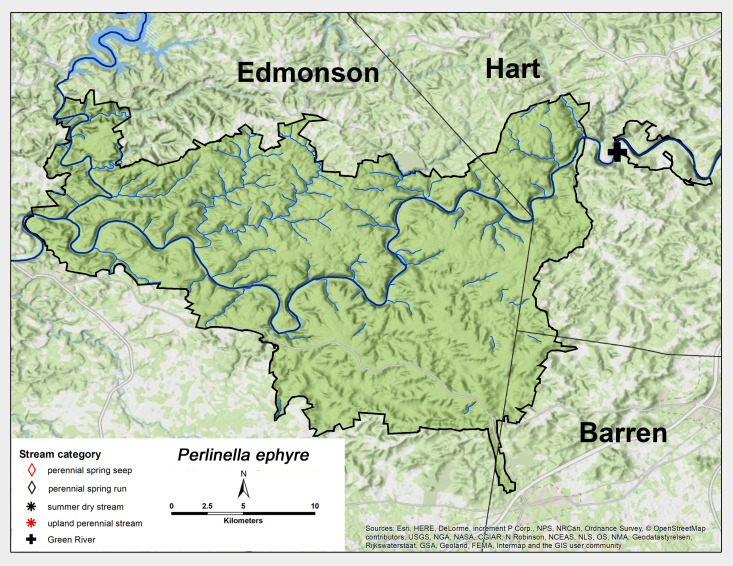


**Figure 13c.**
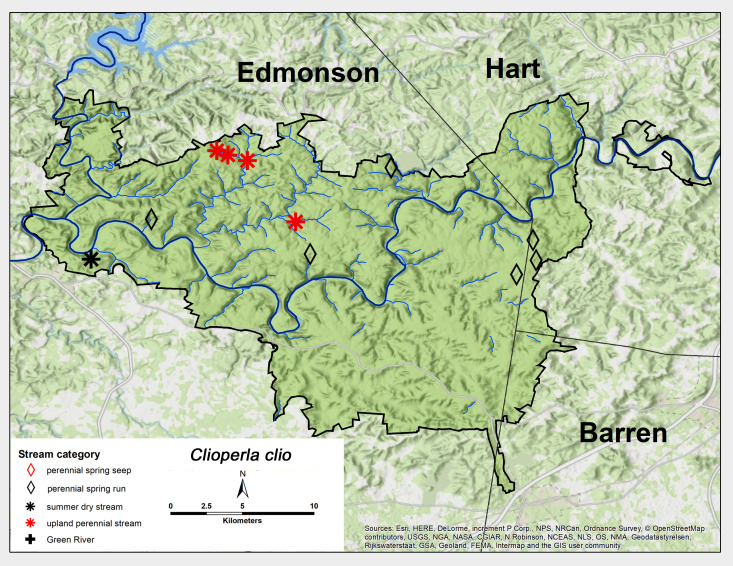


**Figure 13d.**
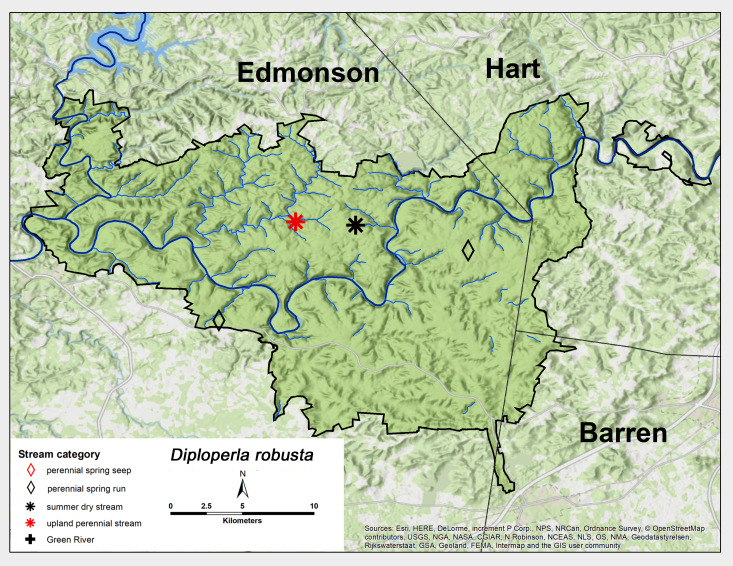


**Figure 14a.**
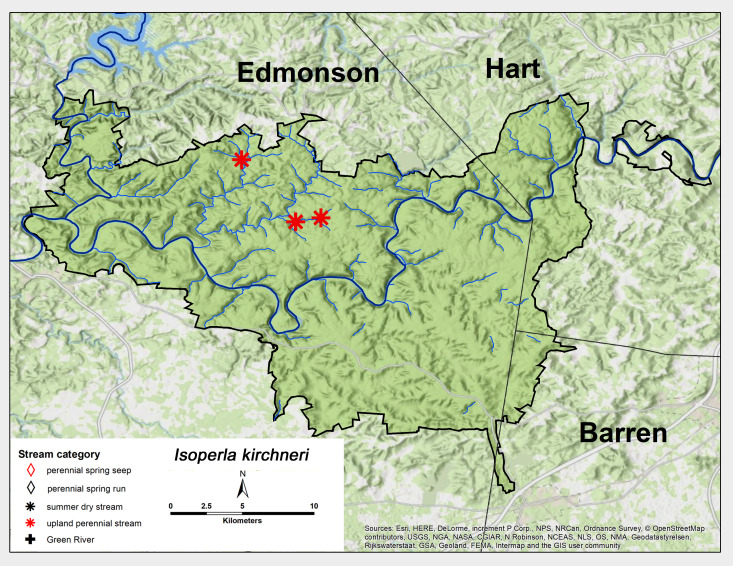


**Figure 14b.**
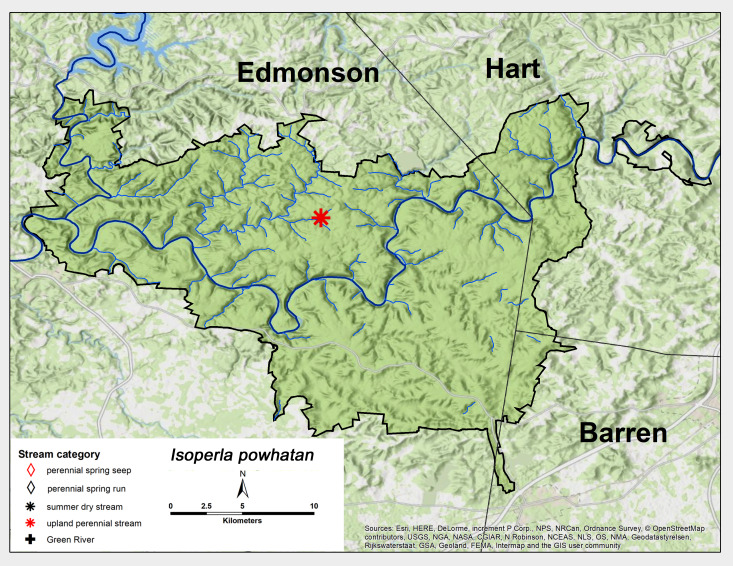


**Table 1.** Collection sites with associated drainage area, coordinates, mean width and flow category. * = sites at the Western Kentucky University Green River Preserve (GRP). DA = drainage area, PSR = perennial spring run, PSS = perennial spring seep, SD = summer dry stream, UP = upland perennial stream.

**Site Name**	**DA (km^2^)**	**Latitude**	**Longitude**	**Width (m)**	**Category**
Green River - Bush Island	4946.9	37.2431	-86.0027	45.4	Green River
Green River - Dennison Ferry	4998.7	37.2168	-86.0492	46.8	Green River
tributary Fishtrap Hollow	0.1	37.2001	-86.1444	1.2	PSR
tributary Green River	0.1	37.2150	-86.2125	2.0	PSR
spring Goodsprings Church	0.2	37.2096	-86.1476	3.2	PSR
tributary Green River	0.2	37.2160	-86.2192	0.9	PSR
tributary Green River	0.2	37.2160	-86.2194	1.9	PSR
Spring out of Silent Cave	0.3	37.1716	-86.1836	1.2	PSR
tributary Green River	0.6	37.2119	-86.2040	2.2	PSR
Collins Spring	<0.1	37.2061	-86.0489	1.5	PSR
Cooper Spring	<0.1	37.1974	-86.0476	1.4	PSR
Adwell Spring	0.2	37.1913	-86.0559	1.8	PSR
Bransford spring	0.3	37.2016	-86.0767	1.5	PSR
tributary Wet Prong	0.1	37.2337	-86.1743	0.6	PSR
Three Springs	0.3	37.1938	-86.0834	1.4	PSR
Blue Spring	0.6	37.2424	-86.1797	1.4	PSR
tributary Ugly Creek	0.4	37.2370	-86.1097	1.0	PSR
tributary Green River	<0.1	37.2102	-86.2131	1.3	PSR
tributary Wet Prong	0.5	37.2421	-86.1752	2.3	PSR
unnamed seep*	0.1	37.2435	-85.9847	seep	PSS
Adwell Spring	0.2	37.1902	-86.0570	seep	PSS
tributary Dry Prong	0.2	37.2099	-86.1584	seep	PSS
tributary Ugly Creek	0.2	37.2389	-86.1119	seep	PSS
tributary Green River	0.1	37.2144	-86.2228	seep	PSS
tributary Sal Hollow	0.1	37.1968	-86.1424	0.5	SD
tributary Buffalo Creek	0.1	37.1997	-86.1557	0.7	SD
tributary Buffalo Creek	0.3	37.2001	-86.1578	0.8	SD
tributary Dry Prong	0.3	37.2097	-86.1640	0.7	SD
tributary Green River	0.3	37.1978	-86.2384	1.7	SD
tributary Mill Branch	0.5	37.2121	-86.1381	0.9	SD
Dry Branch	3.4	37.1975	-86.2272	7.8	SD
Dry Branch	4.2	37.2009	-86.2322	8.1	SD
tributary Sal Hollow	0.1	37.1989	-86.1435	0.7	SD
tributary Big Hollow	0.2	37.2123	-86.1256	0.6	SD
tributary Big Hollow	0.1	37.2073	-86.1248	0.5	SD
Wet Prong	0.4	37.2443	-86.1845	1.8	SD
tributary Wet Prong	0.1	37.2418	-86.1825	0.8	SD
tributary Wet Prong	<0.1	37.2414	-86.1859	0.5	SD
tributary Green River	0.5	37.2170	-86.2247	0.5	SD
Mill Branch	0.5	37.2154	-86.1398	1.4	UP
tributary McCoy Hollow	0.7	37.2119	-86.2040	2.6	UP
tributary Wet Prong	1.6	37.2404	-86.1738	4.8	UP
Mill Branch	2.1	37.2137	-86.1507	5.3	UP
Wet Prong	3.2	37.2400	-86.1713	2.1	UP
Wet Prong	5.6	37.2338	-86.1733	6.3	UP

**Table 2.** List of stonefly species collected at Mammoth Cave National Park and the WKU Green River Preserve (GRP). GR = Green River, PSR = perennial spring run, PSS = perennial spring seep, SD = summer dry stream, UP = upland perennial stream. # = number of unique localities per species, % = of all individuals collected. * = species collected at GRP.

**Family**	**Species**	**Type**	#	%
Capniidae	Allocapnia granulata*	GR	2	1.0
	*Allocapnia mystica*	SD, UP	4	0.8
	*Allocapnia recta*	All	22	27.0
	*Allocapnia rickeri*	PSR, PSS, SD, UP	12	10.0
Leuctridae	*Leuctra alta*	PSS, SD	11	8.9
	*Leuctra rickeri*	SD, PSR, PSS, UP	10	1.2
	*Leuctra schusteri*	PSR, PSS, UP	16	10.0
	*Leuctra sibleyi*	SD, PSS, PSR, UP	20	7.6
	*Leuctra tenuis*	UP	4	2.0
	*Paraleuctra sara*	UP	3	0.4
	*Zealeuctra claasseni*	SD	2	0.1
	*Zealeuctra fraxina*	PSR, SD, UP	3	0.8
Nemouridae	*Amphinemura alabama*	PSR, SD	13	2.0
	*Amphinemura nigritta*	PSR, PSS, SD, UP	20	7.0
	Amphinemura varshava*	PSR, PSS, SD, UP	22	6.0
	*Ostrocerca truncata*	PSR, SD, UP	7	2.0
	*Soyedina calcarea*	PSR, PSS, UP	11	5.0
Taeniopterygidae	*Strophopteryx fasciata*	GR	1	0.1
	Taeniopteryx burksi*	GR, PSS	3	2.0
	Taeniopteryx lita*	GR	1	0.1
	Taeniopteryx maura*	GR	2	0.2
Pteronarcyidae	Pteronarcys dorsata*	GR	2	0.8
Chloroperlidae	*Haploperla brevis*	UP	1	0.1
	*Sweltsa hoffmani*	PSR	2	0.3
Perlidae	*Acroneuria abnormis*	PSR, UP	8	2.0
	*Acroneuria perplexa*	GR	1	0.1
	*Eccoptura xanthenes*	PSS	2	0.1
	*Neoperla stewarti*	GR	1	0.1
	Perlinella drymo*	GR	1	0.1
	Perlinella ephyre*	GR	1	0.1
Perlodidae	*Clioperla clio*	PSR, SD, UP	11	0.8
	*Diploperla robusta*	PSR, SD, UP	3	0.3
	*Isoperla kirchneri*	UP	3	0.8
	*Isoperla powhatan*	UP	1	0.1

**Table 3.** Functional life history traits for stonefly species collected at Mammoth Cave National Park and the WKU Green River Preserve. Volt = Voltinism: 1- or 2-yr development; Devp = Development: 1 = fast seasonal, 2 = slow seasonal; Diap = Diapause: 1 = present, 2 = absent.

**Family**	**Species**	**Volt**	**Devp**	**Diap**
Capniidae	*Allocapnia granulata*	1	1	1
	*Allocapnia mystica*	1	1	1
	*Allocapnia recta*	1	1	1
	*Allocapnia rickeri*	1	1	1
Leuctridae	*Leuctra alta*	1	1	2
	*Leuctra rickeri*	1	1	2
	*Leuctra schusteri*	1	2	2
	*Leuctra sibleyi*	1	1	2
	*Leuctra tenuis*	1	1	2
	*Paraleuctra sara*	1	1	2
	*Zealeuctra claasseni*	1	1	2
	*Zealeuctra fraxina*	1	1	2
Nemouridae	*Amphinemura alabama*	1	1	2
	*Amphinemura nigritta*	1	1	2
	*Amphinemura varshava*	1	1	2
	*Ostrocerca truncata*	1	1	2
	*Soyedina calcarea*	1	2	2
Taeniopterygidae	*Strophopteryx fasciata*	1	1	1
	*Taeniopteryx burksi*	1	1	1
	*Taeniopteryx lita*	1	1	1
	*Taeniopteryx maura*	1	1	1
Pteronarcyidae	*Pteronarcys dorsata*	2	2	2
Chloroperlidae	*Haploperla brevis*	1	1	2
	*Sweltsa hoffmani*	1	2	2
Perlidae	*Acroneuria abnormis*	2	2	2
	*Acroneuria perplexa*	2	2	2
	*Eccoptura xanthenes*	2	2	2
	*Neoperla stewarti*	1	2	2
	*Perlinella drymo*	1	2	2
	*Perlinella ephyre*	1	2	2
Perlodidae	*Clioperla clio*	1	2	1
	*Diploperla robusta*	1	2	1
	*Isoperla kirchneri*	1	2	1
	*Isoperla powhatan*	1	2	1

**Table 4.** Adult presence graph of species collected at Mammoth Cave National Park and the WKU Green River Preserve. Months are noted by Roman numerals and split into halves. Black bars indicate positive collections of adults; grey bars indicate when adults were likely present, but not collected. The latter is based on collection data by the second author from southern and central Kentucky.

	**Month**																							
**Species**	**XII**		**I**		**II**		**III**		**IV**		**V**		**VI**		**VII**		**VIII**		**IX**		**X**		**XI**	
*Leuctra schusteri*																								
*Taeniopteryx burksi*																								
*Taeniopteryx lita*																								
*Taeniopteryx maura*																								
*Allocapnia recta*																								
*Allocapnia rickeri*																								
*Allocapnia granulata*																								
*Strophopteryx fasciata*																								
*Zealeuctra fraxina*																								
*Soyedina calcarea*																								
*Allocapnia mystica*																								
*Zealeuctra claasseni*																								
*Paraleuctra sara*																								
*Amphinemura nigritta*																								
*Amphinemura varshava*																								
*Leuctra alta*																								
*Leuctra sibleyi*																								
*Clioperla clio*																								
*Pteronarcys dorsata*																								
*Amphinemura alabama*																								
*Ostrocerca truncata*																								
*Sweltsa hoffmani*																								
*Isoperla kirchneri*																								
*Perlinella drymo*																								
*Haploperla brevis*																								
*Leuctra rickeri*																								
*Acroneuria abnormis*																								
*Acroneuria perplexa*																								
*Diploperla robusta*																								
*Perlinella ephyre*																								
*Isoperla powhatan*																								
*Neoperla stewarti*																								
*Eccoptura xanthenes*																								
*Leuctra tenuis*																								
